# Complex dynamics of social learning in groups of wild Arabian babblers

**DOI:** 10.1093/beheco/araf099

**Published:** 2025-09-15

**Authors:** Naama Aljadeff, Oded Keynan, Arnon Lotem

**Affiliations:** School of Zoology, Faculty of Life Sciences, Tel-Aviv University, Tel-Aviv 6997801, Israel; Dead Sea & Arava Science Center, Masada National Park, Dead Sea 869100, Israel; Ben-Gurion University of the Negev, Eilat Campus, Eilat 8855630, Israel; School of Zoology, Faculty of Life Sciences, Tel-Aviv University, Tel-Aviv 6997801, Israel

**Keywords:** social learning, foraging behavior, learning dynamics, conformity, cooperative breeding

## Abstract

We studied the effect of a demonstrator on the learning of a novel foraging task in 12 groups of free-living cooperative breeding Arabian babblers (*Argya squamiceps*). We allowed naïve babblers to forage jointly on a foraging grid with a demonstrator previously trained to solve a task in one of 2 possible methods: lifting covers of 1 color or pecking through covers of another color. We found that most group members learned to solve the task using one of the methods, and persisted with it even when later tested with covers of a third (neutral) color that could be opened by both lifting and pecking. However, the method learned by group members did not necessarily follow the method used by the pre-trained demonstrator. Instead, learners within each group tended to use the same method (significantly more than expected by chance), and the extent to which groups differed from the demonstrator was correlated with the extent to which the demonstrator occasionally (and quite rarely) exhibited also the alternative method. These results, together with further analysis of the sequence of events in each group, suggest that both naïve birds and demonstrators learn socially from each other, as well as through individual trial-and-error learning, which enables naïve individuals to become demonstrators themselves and influence the pattern of social transmission. This process mostly leads to a homogenous group behavior, but one that cannot be predicted by the seeded demonstration.

## Teaser

Our study shows that wild Arabian babblers acquire new foraging skills through the combination of social and individual (trial-and-error) learning. These learning processes interact in different ways across groups, leading to the emergence of group-specific behavioral traditions. This highlights how variation in learning dynamics can shape the development of behavioral diversity in animal societies.

## Introduction

Social learning, the process through which innovations or new skills can be transmitted, has been suggested to be a powerful mechanism for sharing information and coordinating behavior within groups (Danchin et al. 2004; Galef and Laland 2005; Shettleworth 2009; Hoppitt and Laland 2013), as well as one of the major benefits of group living (Galef and Giraldeau 2001). Cooperative breeding, a unique social structure in which individuals other than the breeding pair assist in the care of offspring, offers an interesting system for investigating learning strategies and social learning in particular (Burkart 2008). The reason for this is twofold: First, in cooperative breeding species, non-breeding individuals have multiple opportunities to learn from more experienced or knowledgeable group members, thereby enhancing their ability to care for offspring and contribute to the group's overall success. For example, in cooperatively breeding pied babblers (*Turdoides bicolor*), nestlings learn from adults to associate certain vocalizations with food delivery (Raihani and Ridley 2008), and in meerkats (*Suricata suricatta*) pups learn valuable skills such as prey handling from older group members (Thornton and McAuliffe 2006). Second, social learning may be essential for establishing and maintaining cooperative behaviors, as it allows individuals to acquire and transmit the skills and knowledge necessary for successful cooperation. For example, social learning may facilitate cooperative behaviors like mobbing and defending against predators (Thornton and Clutton-Brock 2011), and may thus lead to increased levels of cooperation (Truskanov et al. 2020), ultimately facilitating the evolution of cooperative breeding itself (Earley 2010; Hayashi et al. 2017) (but see Lehmann et al. 2008; Lamba 2014, for a different view).

Yet, how foraging-related information is socially transmitted in cooperatively breeding free-living groups remains relatively unexplored (Langen 1996; Midford et al. 2000; Thornton 2008; Ratikainen et al. 2012). On the one hand, the strong and permanent social ties within cooperative breeders’ groups seem to provide ideal conditions for social learning. On the other hand, competition for food among group members may favor individual specialization rather than social conformity, as suggested for other socially foraging birds (Giraldeau 1984; Aljadeff et al. 2020—see also Borofsky and Feldman 2022, for a recent theoretical analysis). That is, despite being highly social, the relative use of social versus individual (trial-and-error) learning in cooperative breeding birds may also be affected by the level of competition arising when using each of these learning modes.

For example, in a study carried out on a socially foraging species that is not a cooperative breeder (the house sparrow), the behavior of naïve individuals that were introduced into flocks of knowledgeable demonstrators was tested in a competitive setting (ie where foragers competed for a limited amount of food provided in scattered feeding wells). The results of this study showed that naïve individuals did not learn socially the behavior of the demonstrators but rather learned the alternative behavior through individual experience, directing them to feeding wells with lower competition and resulting in distinct foraging specializations within groups (Aljadeff et al. 2020). This study also showed that individuals in such diverse groups could forage more efficiently in heterogeneous environments, suggesting that when animals forage socially on depleted resources, individual learning that facilitates foraging diversity may be more adaptive than social learning that leads to uniformity or conformity (Aljadeff et al. 2020). However, a follow-up study showed that house sparrows tested under the same competitive conditions may develop suboptimal conformity instead of adaptive diversity when the level of task difficulty increases (Marković et al. 2023). These findings inspired the present study that aimed to explore a similar set of questions but in a highly social cooperative breeding species. Specifically, the goal of the present study was to test the extent to which cooperative breeding birds facing a novel foraging task under competitive conditions, would rely on social as opposed to individual learning.

To address this question, we carried out a set of field experiments in a population of free-living Arabian babblers. The Arabian babbler (*Argya squamiceps*) is a cooperative breeding bird species found in arid regions of the Middle East. The babblers commonly live in stable groups with a year-round territory (Zahavi 1989, 1990), and exhibit a relatively complex social system in which groups usually consist of a dominant breeding pair and several subordinate individuals that help raise the young of the group (Lundy et al. 1998). Babblers spend much of their time foraging socially, digging in the ground or under the bark of trees in search of arthropods as well as seeds and soft parts of plants (Zahavi 1990; Kam et al. 2003). The babbler groups tested in this study are continuously monitored as part of the long-term research project at the Shezaf Nature Reserve (Zahavi and Zahavi 1999). They are habituated to human presence, allowing manipulation and close-range monitoring of their social foraging and learning dynamics (Keynan et al. 2015, 2016). Moreover, the results of a previous study that was carried out on the same population suggest that babblers may use social learning during group foraging (Keynan et al. 2016).

To test the extent to which babblers rely on social as opposed to individual learning we presented them with a novel foraging task that can be solved in one of 2 possible methods and with a pretrained group member that can demonstrate only one of these methods. In each babbler group, we first trained the demonstrator to solve the foraging task in one of the 2 possible methods: lifting covers of feeding wells of 1 color or pecking through covers of another color. We then allowed the demonstrator and all other group members to forage jointly on a foraging grid containing multiple rewarding feeding wells of the 2 cover types. This setup allowed the naïve babblers to learn the task of cover opening through either social or individual learning (ie copying the demonstrator or learning the technique irrespective of the demonstrator's behavior). Importantly, both the number of feeding wells and the amount of food provided in each well were limited, inducing competitive conditions and faster food depletion in the cover type used by most individuals and particularly, by the pretrained demonstrator. On the other hand, learning to open the alternative cover type, for which competition was less intense, could have been difficult without social demonstration. We therefore tested whether the naïve individuals copied the demonstrated method and were thus restricted to forage from the demonstrated cover type (as predicted by social conformity, and demonstrated, eg, in great tits [Aplin et al. 2014]), or whether they managed to learn the alternative method asocially, forming groups of mixed specialists or generalists capable of opening both types of feeding wells (as predicted by the skill-pool effect [Giraldeau 1984; Aljadeff et al. 2020]). We further examined whether individuals consistently used the method they learned when tested with feeding wells of a new color that could be opened by both methods (ie by both lifting or pecking).

## Methods

### Study animals and research setup

**Fig. 1.**
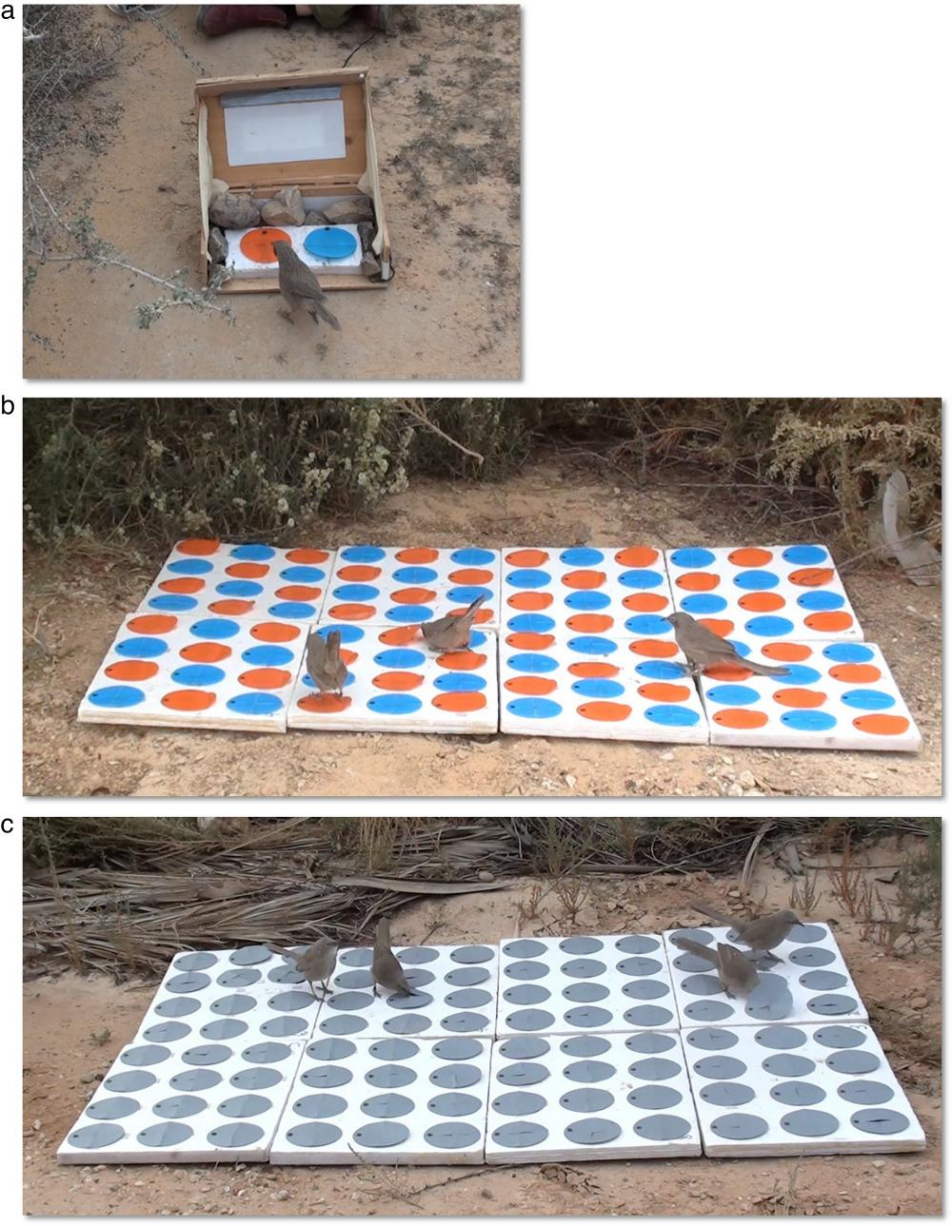
a) Training of demonstrators (Stage 1 of the experiment) was carried out using a foraging box containing 2 feeding wells, one that can be opened by pecking a cover (orange in this example) and the other by lifting a cover (blue in this example). Each demonstrator was trained to open only one of the cover types (see text for further details). b) Social foraging of naïve group members in the presence of a demonstrator (in the center) who was pretrained to lift orange covers (Stage 2 of the experiment). c) Group foraging on a uniform grid of wells with gray covers, all of which can be opened to extract a reward by pecking or lifting (Stage 3 of the experiment). In the photo, 1 babbler (on the right) lifts a gray cover while another (on the left) pecks through a cover.

The study was conducted on free-living Arabian babbler groups of the long-term research system at the Shezaf Nature Reserve, near Hatzeva, Israel. All individuals in the reserve are ringed with a unique combination of 1 metal and 3 colored rings and are habituated to human presence (for a description of habituation, see Zahavi and Zahavi 1999; Ridley 2007), allowing close-range observations and manipulations on social foraging dynamics. The experiments (detailed below) were conducted between June 2018 to Feb 2019 on 12 natural groups of cooperatively breeding babblers that consisted of 4 to 10 individuals, each. Groups included adults and juveniles of both sexes and different social ranks. For the experiments, a soft portable board made of 8 subunits (hereafter: the foraging grid) was assembled in the field and positioned on the ground (Fig. 1). The grid was presented when all group members were present and potentially available to interact with it, typically in central locations within the group's territory, not far from the roost or the breeding site. At the beginning of each session (ie a presentation of the grid) all feeding wells were filled with 1 small mealworm, which was replenished before the next session if consumed. All experimental sessions were recorded using HD video cameras, to enable a detailed analysis of learning processes and social dynamics.

### Experimental design

The experiments included 3 stages that were carried out as follows:

#### Stage 1: pretraining of demonstrators

In the first stage, 1 male in each babbler group was individually trained as a demonstrator for one of 2 cover opening methods; pecking or lifting a soft plastic cover to extract food from a feeding well. Within a group, each foraging method was also associated with covers of a particular color, blue or orange, to enhance the babblers’ ability to discriminate between the 2 methods (color-method associations were counter-balanced across groups). We trained each demonstrator by presenting it with a foraging box, containing 2 feeding wells, one that can only be opened by pecking and the other that can only be opened by lifting (Fig. 1a). Covers designed to be opened by pecking had a slit at the center (in the shape of a cross) through which the bird could peck, but the sides of the cover were attached to the grid, making lifting impossible. Covers designed to be opened by lifting were attached to the surface from 1 side only and had no slit for pecking. By exposing the demonstrators to both types and colors of covers we ensured that they would be accustomed to facing them side by side on the foraging grid. However, we trained the demonstrator to open only one of the cover types. To that aim, we applied the process necessary for training the demonstrator with only one of the cover types. In this shaping (scaffolding) process, the cover was initially presented fully open with the food reward exposed during the first trials, but was gradually presented half-closed, and eventually fully closed until the bird learned to open fully closed covers. We also provided food rewards only under the cover type that was the target of training. All demonstrators were focused on the target cover type and never attempted or managed to open the alternative cover type during the pretraining stage. We trained the demonstrators individually, in the absence of all other group members. Thus, naïve babblers were not exposed to any of the cover opening methods before the beginning of the experiment. To isolate the demonstrator from other group members during the training process, we lured it with food (babblers at our study site are accustomed to being fed by people) to a location behind a bush or a tree, typically 5 to 30 meters away from other group members. The portable training apparatus was visually blocked from 3 sides (see Fig. 1a), and even if another group member approached, the experimenter could quickly close the apparatus using a rope, ensuring that it could be sealed immediately before the approaching individual could observe it. Demonstrators mastered the cover opening method after 10 to 30 presentations of the feeding box over 2 training days when a criterion of 5 consecutive successful openings was reached.

In half of the groups, the demonstrator was trained on lifting the cover, and in the other half, on pecking through the cover (6 vs. 6 groups, respectively). The color associated with each method was fixed within a group but was counterbalanced between groups (in half of the groups blue was associated with pecking and orange with lifting, and in the other half, it was the opposite). Only males were trained as demonstrators since, in some groups, females were occupied with nesting, making their presence less predictable. Half of the demonstrators were dominant males and the other half were subordinates, and they were assigned to demonstrate lifting or pecking in a randomized block fashion (3 dominants and 3 subordinates demonstrated lifting, and 3 dominants and 3 subordinates demonstrated pecking). We used both dominant and subordinate males as it provides an opportunity to test the potential effect of social rank on social learning (Kendal et al. 2015; Canteloup et al. 2020).

#### Stage 2: the effect of demonstrators on the method learned by naïve group members

In the second stage, each group was presented with a mixed foraging grid containing an equal number of feeding wells of each type, distributed as a chessboard array; 96 wells, 48 of each type (Fig. 1b). Importantly, within each group, the color-method association remained as in the training stage of its demonstrator. A foraging session started with all wells on the grid containing 1 small mealworm that, if consumed during the session, was replenished before the subsequent one. Experimental sessions lasted until reaching one of 2 predetermined criteria: either the group had depleted approximately 95% of 1 well type, or 70% of all feeding wells, whichever occurred first. These criteria were chosen to allow sufficient opening attempts and possible competition for the preferred cover type (mean session duration ± std was 14.5 ± 12 min). Groups were given additional sessions until all group members who were active on the foraging grid specialized in at least one of the cover opening methods (4 to 12 sessions in the different groups, and up to 4 sessions per day).

#### Stage 3: testing individual consistency in cover opening behavior

In the third stage of the experiment (Fig. 1c), all groups were presented with a uniform foraging grid in which each well could be opened by lifting or pecking, and all had covers of the same color (ie all 96 feeding wells were covered with gray lids that could be lifted or pecked to extract food). Thus, at this stage, individuals could easily switch their cover opening method or exhibit consistency in their previously learned method. Each well initially contained 1 mealworm and, if consumed, was replenished before the following session. Because, at this stage, the birds were already skilled in cover opening, only 2 relatively short sequential sessions were needed to measure consistency. Each session lasted until 2 or more individuals were active on the grid together for at least 5 min.

### Behavioral and data analysis

We analyzed the video footage of all foraging sessions using 2 Python-based programs developed in our lab: 1. Perspective Birdy, written by Y. Perry and E. Shellef, and 2. Poke-a-bird, written by M. Keren (the second program is simply an improved version of the first one, which became available during the study, see https://arnonlotem.weebly.com/technical-tools--code.html). Using these programs, each bird could be visually tracked throughout its time on the grid and its behaviors could be scored and registered with time and location on the grid into a datasheet.

#### Classifying cover opening actions and defining foraging steps and foraging success

During video analysis, we classified cover opening actions into the following 4 categories: “rewarding lift or rewarding peck”: full action that resulted in food consumption, “nonrewarding” lift or “nonrewarding peck”: full action which did not lead to food extraction (either because the bird failed to extract the mealworm or because the well was previously visited by another bird that extracted it). We defined a foraging step by the focal individual to be any of the actions of the above categories (ie a foraging step can be a rewarding lift, nonrewarding peck, etc.). In cases where several sequential actions were performed on the same well, the sequence was defined as one foraging step, and if it included a rewarding lift or peck, the entire foraging step was classified as rewarding. Otherwise, it was classified as nonrewarding (lift or peck). The rationale behind this classification is that a sequence of interactions with the same foraging well was sometimes needed to extract the mealworm reward. The distinction between rewarding and nonrewarding steps was made to control for the possibility that 1 method was, in practice, more rewarding than the other. Such bias could emerge either because extracting the mealworm was easier using one of the methods, or because the associated cover type was less commonly used by other group members, increasing the chance of finding rewarding wells of this type. For individuals who used both methods, we could thus measure which of the methods was experienced as more rewarding and whether this experience can explain the preference for 1 method over the other.

#### Scoring cover opening behavior

Cover opening behavior was scored as the proportion of lift actions out of the total number of actions (ie number of lifts/(number of lifts + number of pecks). Accordingly, a bird that always opens the covers by lifting will have a score of 1, a bird that always pecks through the cover will have a score of zero, and a bird that uses both methods equally should have a score of 0.5. Because each score reflects the actual counts of lifts and pecks, we could analyze cover opening behavior as a binomial variable using these raw counts (see Statistical analysis below). To ensure a reliable score for a focal individual, we excluded individuals who performed less than 6 foraging steps (ie 6 cover openings) throughout the entire experiment. This criterion was determined a priori following previous studies, as 6 is the minimal number of choices required to detect a significant preference using a binomial test (see also Ilan et al. 2013; Truskanov et al. 2018; Aljadeff et al. 2020). We also used another form of cover opening score in which instead of scoring the proportion of lift actions we scored the proportion of copying the demonstrator's method (the proportion of correct copying). That is, for babblers whose demonstrator was a lifting demonstrator, the score was the same as the proportion of lifts, but for babblers whose demonstrator was a pecking demonstrator, it was the inversed proportion (ie the proportion of pecking). This score allowed us to combine data from the 2 experimental groups (lifting and pecking), providing a single measure of each individual's tendency to copy the demonstrator (see Results).

#### Measuring demonstration behavior

While the experimental design aimed to provide only 1 type of action demonstration for each group, it was important to confirm that this was indeed the behavior exhibited by the pretrained demonstrator and, if not, to assess how variation in demonstration behavior affected learning dynamics. Quantifying the demonstrator's effect is complicated by the fact that different group members spent varying amounts of time with the demonstrator on the foraging grid. Therefore, for each learner we extracted all demonstrations it could potentially observe during the time it spent on the grid and calculated the lift proportion of these demonstrations. We could then analyze the learning performance of individual learners (ie their lift proportion) in relation to this estimate. We also considered the possibility that each focal learner could be affected by the behavior of other naïve group members who had learned the task from the pretrained demonstrator, or through trial-and-error, and could thus act as “secondary demonstrators” to their peers. Since we monitored the cover opening behavior of all group members (see above) we could use their cover opening scores for testing these effects (see Statistical analysis below).

### Statistical analysis

We analyzed the results primarily using Generalized Linear Mixed Models with “lift proportion or the proportion of correct copying” (ie lifting when lift was demonstrated and pecking when peck was demonstrated) as the dependent variables. “Babbler group” was included as a random effect, while “experimental group” (lift versus peck) and other potential factors influencing social learning were included as fixed effects (including demonstrator behavior, demonstrated color, demonstrator's rank, group size, and learner's age, sex, and rank). Lift proportion and the proportion of correct copying were modeled as binomial response variables using the actual counts of lifts and pecks, or correct and incorrect copying (eg R syntax: glmer(cbind(number_of_lifts, number_of_pecks) ∼ experimental group + (1 | group), family = binomial, data = data_filename). To distinguish between within-group and between-group effects of demonstrator behavior we used group-mean centering, as described in previous work (Enders and Tofighi 2007; Van de Pol and Wright 2009). Specifically, we included both group-level means (representing between-group effects) and individual deviations from those means (within-group effects) as fixed effects in the GLMM (see Results for details). Additionally, we ran permutation analyses to compare the observed similarity in cover opening behavior within babbler groups to that expected by chance (see Results). To assess whether group behavior was shaped primarily by the pretrained demonstrator or by secondary demonstrators, we repeated these analyses both with and without the pretrained demonstrators, testing which condition—presence or absence of the pretrained demonstrator—resulted in greater behavioral similarity within groups. All analyses were performed in R v. 4.4.0 (R Development Core Team 2024).

## Results

### The behavior of the pretrained demonstrators

During Stage 2 of the experiment, the pretrained demonstrators foraged jointly with the naïve group members and demonstrated almost exclusively the cover opening method that they were trained to use (see Figure S1 and statistics therein). The pretrained demonstrators were active on the grid, allowing naïve individuals to observe their cover opening method and interact with the foraging wells in their presence (see photo in Fig. 1b). All naïve individuals exhibited overlapping social foraging times with the pretrained demonstrator and with other naïve individuals (in groups with multiple participating naïve babblers). The mean time duration spent with the pretrained demonstrator did not differ from the mean duration spent with other naïve individuals (see Figure S2 and statistics therein), suggesting that learning opportunities from the pretrained demonstrator and other group members were similar (though the pretrained demonstrator could demonstrate right from the start while other group members could become secondary demonstrators only after they learned to open covers).

### Learning the cover opening methods by naïve group members: the effect of the pretrained demonstrators

**Fig. 2.**
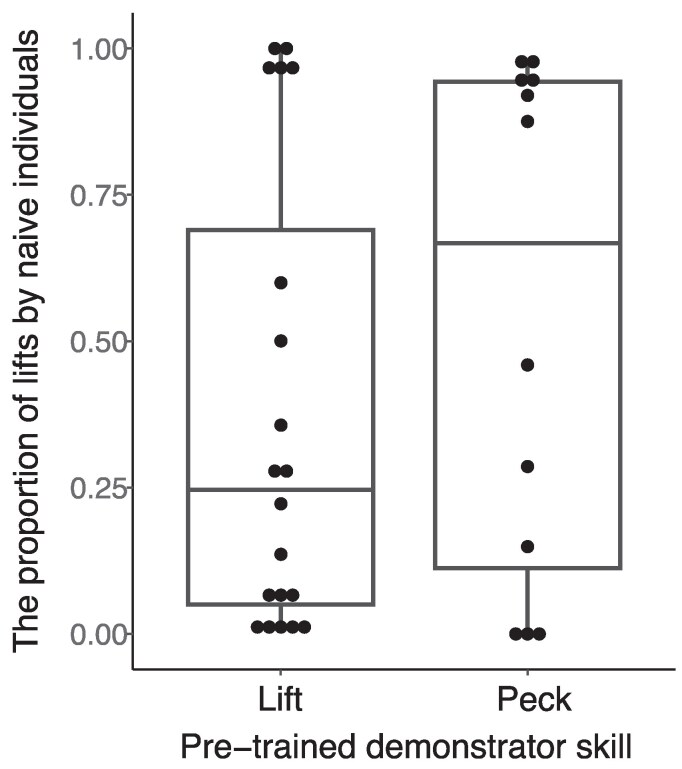
The proportion of lift actions out of all actions (lifts and pecks) performed by naïve group members of the lift and peck demonstration groups during all sessions of Stage 2. Data are represented as data points, median, 25%, and 75% quantiles.

During Stage 2 of the experiment, 44 out of 52 naïve individuals from the 12 groups successfully learned to open covers using at least one of the cover opening methods (see Table S1). Among these, 32 individuals opened at least 6 covers, enabling their inclusion in the analysis (see Methods). Twenty of these individuals were from the 6 groups whose pretrained demonstrators used the lift method, and the other 12 were from the 6 groups whose demonstrators used the peck method. The results (Fig. 2, Table 1) show that there was no difference in the proportion of lift actions between the 2 experimental groups. In both groups, most birds tended to specialize in one of the methods (either lift or peck; scores close to 1 or zero, respectively), regardless of the pretrained demonstrator's method. If anything, the lift proportion was a bit higher in the peck demonstration group (see Fig. 2). Thus, there was no indication that the cover opening method used by the pretrained demonstrator affected the method learned by group members.

**Table 1.** Generalized Linear Mixed Model (GLMM) testing how learners’ lift proportions were affected by experimental group (lift versus peck demonstration).

N	Response variable	Fixed effects	Estimate	Std. error	*z* value	*P* value	95% CI (lower, upper)
32	Lift proportion by naïve individuals	(Intercept)	−1.057	1.178	−0.898	0.369	(−3.60, 1.49)
		Demonstrator skill (peck)	1.575	1.679	0.938	0.348	(−2.18, 5.12)

We further examined whether variation in cover opening behavior among naïve babblers could be explained by differences in the demonstrators’ behavior, beyond their general classification to the “lift” and “peck” experimental groups. To that aim, we used a GLMM that included 3 fixed effects. The first was the experimental group. The second captured the between-group effect by including the group-level mean of the demonstrator's lift proportion, reflecting that different groups had different demonstrators who may have varied in their typical use of lifting. The third fixed effect tested the within-group effect, based on each individual's deviation from the group mean of observed demonstrator lift proportion. This allowed us to account for the fact that different group members have spent different amounts of time with the pretrained demonstrator, and thus were exposed to different proportions of lifting behavior. Notably, including all 3 effects allowed us to test the impact of fine-scale variation in demonstrator behavior (the second and third fixed effects) within the broader experimental group classification (the first fixed effect).

**Fig. 3.**
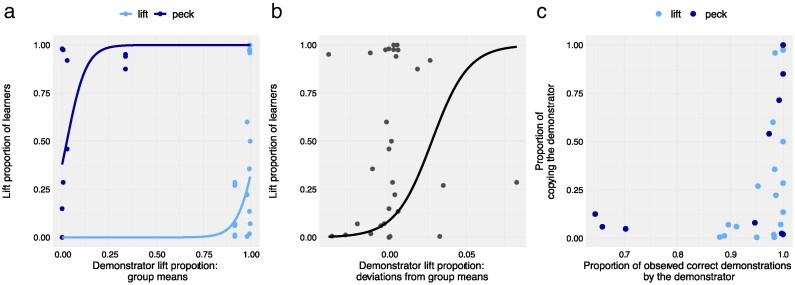
Regression plots illustrating the relationship between demonstrator behavior and learner performance. a) Between-group effects from Model A (Table 2), showing the relationship between group-mean demonstrator lift proportion and learner lift proportion, plotted separately for the lift and peck groups. b) Within-group effect from the same model, showing the relationship between individual deviations from the group mean in demonstrator lift proportion and learner lift proportion. c) Relationship between the proportion of correct copying by naïve birds and the proportion of correct demonstrations made by the pretrained demonstrator when foraging together with the focal naïve bird (see Model B in Table 2).

The results of this analysis (Table 2, Model A) indicate that all 3 effects were significant, with the within-group effect being the most statistically robust. At first, these findings seem to contradict our earlier results (Fig. 2), which suggested that the behavior of the pretrained demonstrator did not influence the learners’ cover opening method. However, the regression plots from the model (Fig. 3a and b) help to clarify this apparent contradiction: although there were both “peckers” and “lifters” within each experimental group (see the far left and right sides of Fig. 3a), which is consistent with Fig. 2 above, within each experimental group, naïve birds who exhibited the opposite behavior from their demonstrator were more likely to belong to groups in which the demonstrator occasionally (and even quite rarely) used also the alternative method (eg a pecking demonstrator with ∼0.3 lifting proportion instead of zero, or a lifting demonstrator with ∼0.1 pecking proportion instead of zero—see Fig. 3a). Moreover, this effect was especially pronounced within groups (Fig. 3b). That is, group members who were exposed to slightly more lifts or pecks than their peers (by as little as ∼4% above or below the group mean) were much more likely to become lifters or peckers, respectively (Fig. 3b; Table 2, Model A).

**Table 2.** Results of GLMMs testing the effects of experimental group (lift vs. peck), and between-group and within-group variation in demonstrator behavior (based on group means and deviations from those means; see text and Methods for details).

Model	*N*	Response variable	Fixed effects	Estimate	Std. error	*z* value	*P* value	95% CI (lower, upper)
A	32	Lift proportion by naïve individuals	(Intercept)	−20.172	9.538	−2.115	0.034	(−40.79, −0.35)
			demonstrator skill (peck)	19.680	9.013	2.183	0.029	(0.87, 39.04)
			Observed lift proportion of demonstrator: Group means	19.429	9.644	2.014	0.044	(−0.61, 40.29)
			Observed lift proportion of demonstrator: Deviations from group means	84.551	5.895	14.344	<0.001	(73.28, 96.37)
B	32	Copying success of demonstrator by naïve individuals	(Intercept)	−20.172	9.343	−2.159	0.031	(−40.79, −0.35)
			demonstrator skill (peck)	1.236	1.750	0.706	0.480	(−2.45, 5.18)
			Observed lift proportion of demonstrator: Group means	19.429	9.449	2.056	0.040	(−0.94, 40.29)
			Observed lift proportion of demonstrator: Deviations from group means	84.551	5.884	14.370	<0.001	(73.28, 96.37)

Model A tests the effect of demonstrator lift proportion on the lift proportion of learners. Model B tests the effect of the proportion of correct demonstrations on the proportion of correct copying by learners.

Another way to illustrate and analyze these effects, is to combine the data of the 2 experimental groups and to score the naïve learners’ behavior as the proportion of copying the pretrained demonstrator (ie the proportion of “lifting” in the lift group, and proportion of “pecking” in the peck group), and to score the behavior of the pretrained demonstrator as the proportion of correct demonstrations (ie the proportion of pecking made by a demonstrator in the peck group, or the proportion of lifting by a demonstrator in the lift group, while foraging together with each focal naïve bird). Plotting these scores, the first against the other, shows that copying proportion is indeed related to a high proportion of correct demonstrations (Fig. 3c). An analysis of this relationship (Table 2, Model B) shows a significant between-group effect as well as a highly significant within-group effect. These results are similar to those of the previous analysis that was based on lift proportions (Fig. 3a and b; Table 2, Model A), but may be more intuitive and easier to grasp as they are focused on the simple relationship between correct demonstration and copying, regardless of lifting and pecking. The only difference between the 2 analyses is that the effect of experimental group that was significant in Model A, was no longer significant when the 2 actions (lifts and pecks) were combined to “correct copying” (in Model B). This may be expected since now both lifters and peckers are measured on the same scale and both have high copying proportions distributed mostly above the 95% range (compare Figs 3a and 3c).

**Fig. 4.**
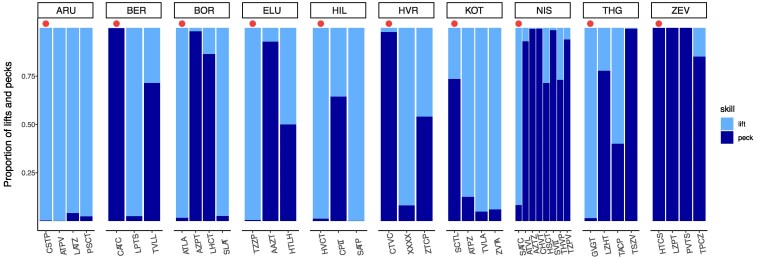
The proportion of lifts and pecks performed by the babblers of 10 groups during Stage 2 of the experiment (see text for further details). The left bar of each group represents the pretrained demonstrator (under the red dots).

It is important to note that the observed relationship between copying and correct demonstration (Fig. 3; Table 2) may reflect not only the influence of the demonstrator on naïve birds, but also the influence of some naïve birds on the demonstrator. That is, some naïve individuals may have learned the alternative method through trial-and-error, which was then copied by the demonstrator. To examine which scenario is more likely, we carefully checked the sequential order of cover opening events in the 8 groups (out of the 12) where naïve birds behaved differently from the demonstrator (ie groups with copying score of <0.5). In 3 of these groups (2 from the lift experimental group, and one from the peck experimental group) it was clear that the demonstrator was the first to open covers using the alternative method before any of the naïve birds learned to open covers. Thus, in these groups, it is quite possible that the few alternative demonstrations affected 1 or more naïve members, who became secondary demonstrators for the rest of the group. However, in 4 of these groups (2 from each experimental group), at least 1 naïve bird learned the alternative method before the demonstrator began using it. Thus, in these groups, the demonstrator may have learned the alternative method from the naïve individuals. Finally, there was 1 group in which the demonstrator never used the alternative method, but the naïve birds did. (This group was not included in the analysis of Fig. 4 below because only one of its 4 naïve birds opened more than 5 covers, but overall, 3 of the 4 naïve birds of this group used the alternative method without being able to learn it from the demonstrator). Altogether, the sequential order of events suggests that the relationship between copying proportion and the proportion of correct demonstrations emerged from social learning by both naïve babblers who learned from demonstrators and by demonstrators who learned from naïve babblers.

### Behavioral similarity within groups: the effect of secondary demonstrators

The effect of social learning is also suggested by the pattern of within-group similarity in cover opening behavior. Figure 4 shows the proportion of lifts and pecks performed by babblers from 10 groups during Stage 2 of the experiment. In all these groups, at least 2 naïve individuals learned to open covers repeatedly (at least 6 times) and, therefore, could potentially influence each other (30 individuals from the 10 groups passed these criteria and are shown in the Figure). The leftmost bars in each group (marked with a red dot) represent the pretrained demonstrators. A visual inspection of Fig. 4 suggests that, although group members may not behave like their pretrained demonstrators (as already illustrated in Fig. 2), they do tend to behave similarly to one another.

To test whether within-group similarity is indeed higher than expected by chance, we conducted a permutation test to generate the expected null distribution of within-group differences. We randomly assigned the lift proportion scores of individuals into simulated groups with the same group sizes as in the original experiment. We then summed up the distances between the lift proportion scores of all group members (or of only naïve individuals) of each simulated group (eg if the scores of 3 group members were 0.7, 0.8, and 0.9, respectively, the sum of distances between them was 0.1 + 0.1 + 0.2 = 0.4). This process was repeated for 10,000 iterations, creating a null distribution against which we could compare the actual group distances.

A comparison of these simulated group distances to the observed group distances revealed that within-group distances of the actual babbler groups were significantly smaller than those of the simulated groups. This result was significant even when the pretrained demonstrators were included in the analysis (Figure S3a, estimated *P* = 0.0143), but highly significant when they were excluded, and only the similarity among naïve group members was examined (Figure S3b, estimated *P* = 0.0001). These findings suggest that within-group similarity in task solution occurs primarily among naïve learners rather than across all group members (including the pretrained demonstrators), which is also consistent with the observations in Fig. 4. Accordingly, most naïve birds learned socially to open covers from formerly naïve group members who became secondary demonstrators rather than from the pretrained demonstrators.

### Testing the potential effect of additional variables on social learning

We explored a range of additional variables that could potentially affect learning dynamics by incorporating them into the GLMM analysis. We used the structure of Model B from Table 2 as the base for this analysis: a GLMM with group as a random effect, and experimental group, group means of correct demonstration proportion, and the deviations from these means, as fixed effects. The dependent variable was the copying proportion, modeled as a binomial outcome. We included the following additional fixed effects in the model to assess their potential influence on learning: learners’ social rank (subordinate vs. dominant), age (in years), sex (male vs. female), group size, demonstrator's social rank (subordinate vs. dominant), demonstrated color (blue vs. orange), and time spent with the demonstrator on the foraging grid. All factors were initially included in the model (Table S2, Model A) and then nonsignificant terms were removed stepwise, resulting in a final model with only significant predictors (Table S2, Model B). The results showed that, alongside the effect of the proportion of correct demonstrations, copying was more likely when learners were subordinates, older, and spent less time with the demonstrator (see Figure S4 for predicted values and regression plots). Notably, while social rank and age are fixed traits during the experiment, time spent with the demonstrator may reflect a consequence rather than a cause of using the alternative method (see Discussion).

We also examined whether individuals’ relative use of lifting versus pecking was related to their relative success with each method, measured as the proportion of actions that successfully retrieved a mealworm. This analysis focused on 14 individuals who used both methods at least 6 times each. For these individuals, we calculated the difference in success rates (proportion of rewarded lifts minus proportion of rewarded pecks) and tested its effect on lift proportion using a GLMM. The results (Table S3; Figure S5) show a highly significant effect: individuals tended to use more frequently the method with which they experienced greater success. It should be noted, however, that the higher success rate may not be the cause of strategy choice, but rather a consequence of individuals becoming more skilled with the method they use more often (see Discussion).

### Individual consistency in cover opening behavior

**Fig. 5.**
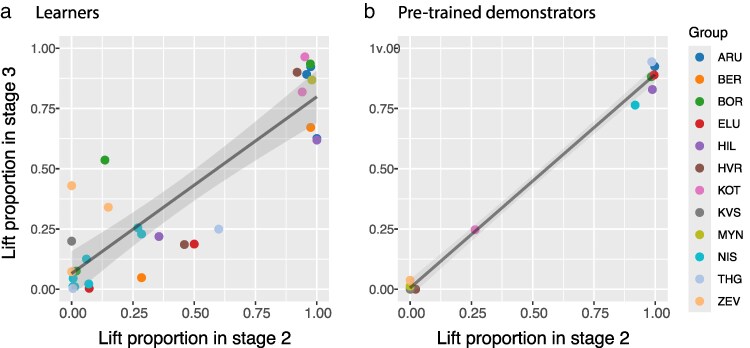
Behavioral consistency of learners and pretrained demonstrators assessed through the relationship between their lift proportion during the 2 sessions of Stage 3 (gray covers that can be opened by both lifting and pecking) and their lift proportion during Stage 2. a) The 30 naïve individuals that opened at least 6 covers during Stage 2 as well as during Stage 3. Colors represent the group to which the babblers belong. The linear trend and confidence interval around it are shown as a black line and a gray area, respectively. b) The same as in (a) but for the 12 pretrained demonstrators.

In Stage 3 of the experiment, when tested with a uniform grid of gray covers that could be opened by both pecking and lifting (see Fig. 1c), the naïve individuals tended to use the same method they learned to use during Stage 2 (Fig. 5a). This consistency was expressed at both the between- and within-group levels (see GLMM results in Table 3). Thus, despite lacking the learned association with the color cue of the cover, babblers showed consistency in their cover opening behavior. Similar (or even slightly higher) consistency was exhibited by the pretrained demonstrators who foraged together with the rest of the group also during Stage 3 (Fig. 5b; *n* = 12, Spearman's rho = 0.851, *P* < 0.0005).

**Table 3.** Results of a GLMM testing whether naïve individuals maintained their learned method in Stage 3. Lift proportion in Stage 3 was predicted by group means (between-group effect) and deviations from group means (within-group effect) in lift proportion during Stage 2, indicating behavioral consistency at both levels.

N	Response variable	Fixed effects	Estimate	Std. error	*z* value	*P* value	95% CI (lower, upper)
30	Lift proportion by naïve individuals in stage 3	(Intercept)	−2.553	0.614	−4.157	<0.001	(−3.88, −1.25)
		Lift proportion in stage 2: Group means	3.953	1.151	3.433	<0.001	(1.5, 6.43)
		Lift proportion in stage 2: Deviations from group means	5.644	0.204	27.713	<0.001	(5.25, 6.05)

## Discussion

In this study, 12 groups of free-living cooperative breeding Arabian babblers were given a novel foraging task that can be solved in 2 different ways and were exposed to a pretrained group member who demonstrated one of these task solutions (pecking through covers of 1 color or lifting covers of another color). Our results show that almost all babblers learned to solve the task with a consistent preference for 1 task solution, but it was not necessarily the solution used by the pretrained demonstrator. Yet, individual choice of task solution was not independent of other group members, and was still affected by the demonstrator's behavior. First, learners of the same group tended to use the same method significantly more than expected by chance. Second, the extent to which groups and individuals within groups differed from the demonstrator was related to the extent to which the demonstrator occasionally (and even quite rarely) displayed the alternative method when observed by those group members. These results suggest that learners were affected by their peers, which is consistent with social learning. However, the pattern of information transfer was not as simple as anticipated. Namely, the behavior learned by most group members was not necessarily the seeded one. In the following, we first discuss the extent to which our data is best explained by social learning, as well as by other possible factors. We then consider the role of competition and task difficulty in producing the observed results, and the role of learning dynamics and chance events in the acquisition of novel task solutions by social groups.

### The evidence for social learning

Our study was based on a simple version of the 2-action experiment, commonly used to provide evidence for social learning (Dawson and Foss 1965; Akins and Zentall 1996; Voelkl and Huber 2000; Heyes and Saggerson 2002; van De Waal et al. 2015). The rationale behind a 2-action experiment is that if social learning occurs, naïve individuals will preferentially copy the demonstrated technique rather than choose randomly between 2 equally effective alternatives (the demonstrated and the alternative technique). This should produce within-group similarity, and possibly, between-group variation in behavior due to different demonstrations in different groups. Traditional 2-action experiments also include a control group with no demonstration. We did not include such a control treatment, because our main goal was to explore the social learning dynamic rather than to demonstrate that the task cannot be learned asocially. Including a control group would have been costly in terms of sample size, and the number of closely monitored groups in a field study of a cooperative breeding species such as the Arabian babbler is normally limited. However, the main challenge to our experimental results is the lack of significant difference between the 2 action groups (lift and peck; Fig. 2). This leaves us mainly with correlative rather than experimental evidence. That is, our data present strong evidence for behavioral similarity within groups but the differences between groups were not determined by the randomized experimental design. Demonstrating social learning in such cases may still be possible by using Network-Based Diffusion Analysis (Hasenjager et al. 2021), which relates transmission patterns to variation in social ties. However, this method is not effective with small, socially cohesive groups, such as our babblers. Consequently, one could argue that the behavioral similarity within babbler groups was not driven by social learning, but rather reflected a scenario in which group members of each group had a pre-existing inclination to use the same method (lifting or pecking) due to factors such as genetic background or specific habitat conditions (eg Vijayan et al. 2007). Even the significant relationship between the demonstrators’ proportion of correct demonstrations and their groups’ mean copying proportion (Table 2, Model B) could be explained by demonstrators trained to behave against their group's pre-existing preference, resulting in more demonstration errors. However, the highly significant within-group effect of demonstrator behavior (Table 2; Fig. 3b) is inconsistent with this alternative explanation. A pre-existing group-level inclination cannot account for why, within each group, poorer copying was associated with individuals exposed to poorer demonstrations (or with demonstrators performing poorly in the presence of group members using the alternative method). Thus, this within-group effect provides strong evidence supporting social learning.

Moreover, the group pre-existing inclination explanation is far less probable than the social learning explanation for several reasons. First, the dispersal dynamic of young babblers (Zahavi and Zahavi 1999; Ostreiher et al. 2023) makes genetic specialization of pecking and lifting behaviors at the level of groups extremely unlikely. Second, nongenetic specialization due to habitat conditions is also unlikely, certainly not to the extent that can explain the observed dichotomy between groups. Most group territories in our study include diverse types of habitats represented in other group territories (see Lundy et al. 1998), and babblers in all groups use both pecking and lifting repeatedly in their daily foraging activity when they peck in the sand, lift various objects, or peck and lift parts of tree barks in their search for insects (Ben Mocha et al. 2024). Additionally, the geographic distribution of groups that use lifting versus pecking seems quite random (see Figure S6). Social learning, on the other hand, has been clearly and repeatedly demonstrated in socially foraging birds (reviewed by Lefebvre and Bouchard 2003; Slagsvold and Wiebe 2011; Aplin 2019), as well as in babblers (Raihani and Ridley 2008), and its existence in our study population was indicated in a previous study (Keynan et al. 2016). Thus, considering this evidence and especially the strong within-group effect discussed above, we can safely conclude that our results reflect the effect of social learning.

### The effect of additional variables on social learning

Individuals’ propensity to learn from others may be affected by sex (Bono et al. 2018), age (Coelho et al. 2015), and social rank (Kendal et al. 2015; Canteloup et al. 2020). Including these factors in our GLMM analysis (Table S2) did not reveal an effect of sex, but revealed significant effects of rank and age—subordinate and older individuals were more likely to copy the primary behavior of the demonstrator (Table S2; Figure S4a,b). Subordinates’ greater inclination to copy others is in line with previous studies (eg Thornton and Malapert 2009; Aplin et al. 2013). The age effect, on the other hand, is less clear because subordinates are on average younger. The model's results suggest that within each social rank class (eg within subordinates), older individuals are more likely to copy, a finding that warrants further study. Copying was also not affected by group size, social rank of the demonstrator, or by the color of the demonstrated covers (Table S2, Model A). Interestingly, however, copying proportion was significantly higher among group members that spent less time with the demonstrator on the foraging grid (see Table S2; Figure S4c). One way to explain this result is to postulate that individuals who spent more time with the demonstrator were more likely to observe them using the alternative cover-opening method, which could have led them to copy the alternative method rather than the primary one demonstrated. While we cannot rule out this possibility, we believe its influence is not strong enough to account for the results, especially given that, in 4 of the groups, some learners began using the alternative method before the demonstrator did (see Results). In our view, a simpler and more plausible explanation is that individuals who learned the alternative method did not face competition from the pretrained demonstrator and were therefore able to stay on the grid longer with them, opening feeding wells of the alternative type. Thus, the effect of “time spent with the demonstrator” was likely a consequence of learning, rather than its cause.

Similarly, the fact that individuals were more successful at retrieving mealworms when using the method they employed more frequently (Table S3; Figure S5) is likely due to becoming more skilled with their chosen method, rather than a choice based on greater initial success. However, regardless of whether this “chicken-and-egg” problem can be resolved, the higher success rate with the initially chosen method may contribute to the development of the behavioral consistency observed in the babblers during stage 3 of the experiment.

### Group diversity versus conformity: the effects of competition and task difficulty

The initial goal of our study was to test whether babblers facing a novel foraging task under competitive conditions would rely on social as opposed to individual learning (see Introduction). We hypothesized that the limited number of feeding wells on the foraging grid, and the fact that each well contains only 1 food item would make social learning disadvantageous (as opposed to individual learning) because it would allow the babblers to learn to open only the demonstrated cover type and, therefore, to potentially access only half of the food available on the grid. Our hypothesis was inspired by a previous theory (Giraldeau 1984) and experimental work with house sparrows (Aljadeff et al. 2020), showing that under competitive conditions, individual learning may be favored because it allows different individuals to utilize different types of food patches rather than conform to 1 behavior only. However, as we mentioned in the introduction, a follow-up study showed that house sparrows tested under the same competitive conditions may develop suboptimal conformity instead of adaptive diversity when the level of task difficulty increases (Marković et al. 2023). This recent study is highly relevant to the experimental results of the present study because in both studies naïve birds had to learn to open covers of feeding wells, which seems to be difficult to learn without social demonstration or gradual shaping. The babblers in our study did not conform to the seeded behavior as the sparrows in Markovic et al. (2023) but they also ended up creating uniform groups, apparently as a result of learning socially from each other. We have evidence that in at least 5 groups, some naïve individuals opened covers using a different method than the demonstrated one, indicating that the task in our study could still be solved through individual trial-and-error learning. However, despite these occasional innovations, it seems that it was easier for most other group members to copy either the primary or the secondary demonstrators rather than to innovate, which explains group similarity.

In previous studies of our research group (Aljadeff et al. 2020; Marković et al. 2023) we suggested that the extent to which socially foraging groups develop diversity in skill acquisition as opposed to conformity may depend on both competition and task difficulty, with competition and tasks that are easy to learn asocially leading to diversity (eg Aljadeff et al. 2020) while reduced competition and tasks that are easier to learn socially lead to conformity (eg Van de Waal et al. 2013; Aplin et al. 2014; Gunhold et al. 2014, 2015; Marković et al. 2023). Our present study suggests an interesting intermediate state where task difficulty is high enough to create mostly uniform groups despite competitive conditions, but is not high enough to restrict the entire group to the seeded behavior (as in Aplin et al. 2014; Marković et al. 2023). That is, in some cases the demonstrator, or one of the naïve individuals, managed to learn the alternative task solution, and this alternative solution, rather than the seeded one, was the behavior that was eventually copied by the rest of the group. In the following subsection, we will discuss how this could have happened despite the initial rarity of demonstrations of the alternative solution, and under what conditions this type of social learning dynamics may be expected.

### Learning dynamics of novel foraging tasks in social groups

Our study suggests that under some conditions, the process of acquiring novel task solutions by social groups may depend on the learning dynamic of some specific individuals, and perhaps on some stochastic or chance events that can shift the entire process in 1 direction or another. In 3 babbler groups in our study, the demonstrators learned by themselves to open covers also by the other method than the one they were trained on and could therefore demonstrate it, though they did so only occasionally and quite rarely (see Fig. 3). Despite the rarity of these demonstrations, 1 or a few naïve individuals learned this method (rather than the commonly demonstrated one) and, once they did, could have demonstrated it repeatedly to other group members, leading the entire group to adopt it. A similar process occurred in 5 babbler groups where one of the naïve birds (rather than the demonstrator) was the first to learn the alternative, nondemonstrated method, which again, allowed all other group members to copy this alternative method rather than the seeded one (see Results).

The fact that in some cases group members learned the relatively rare innovation rather than the behavior that was repeatedly demonstrated by the pretrained demonstrator may be explained by the circumstances that have led the first naïve birds to learn this alternative innovation. The mechanisms responsible for biased transmission, where 1 cultural trait is more attractive or successful than another, have been the subject of extensive theoretical and conceptual discussion (eg Claidière and Sperber 2007; Buskell 2017; Mesoudi 2021). Broadly speaking, these mechanisms can be classified into 2 categories: those involving attentional or perceptual biases (Heyes 2012; Lotem and Halpern 2012; Greggor et al. 2017) and those involving production biases (Chimento et al. 2022; Chimento and Aplin 2024; Toji et al. 2024). Simply put, in the first case, a trait may not be acquired or transmitted because the animal fails to learn it. In the second case, the animal may learn the trait but is less likely to perform it, either due to difficulty in execution or a predisposition to favor certain actions over others. These 2 classes of mechanisms are not mutually exclusive, and both may contribute to, or explain our results. Learners may not necessarily pay attention to all demonstrations alike but may be affected by particular demonstrations that were more salient than others or more effective in some other ways (proximity, viewing angle, observer's attention at a particular moment, etc.). Alternatively, some individuals may have recognized there was a mealworm under the cover and noticed the demonstrated action. However, they might have attempted to emulate the action's outcome using their own preferred actions, leading them to peck rather than lift, or vice versa. Moreover, learners who tried to copy the demonstrated action but selected the wrong cover type (and failed) may have discovered the alternative method through trial-and-error (see Truskanov and Lotem 2017, for a similar process). Importantly, regardless of the underlying causes, once an innovator (or an individual who learned from the demonstrator's rare innovation) starts using the newly acquired method, demonstrations of this newly acquired behavior may become as common as those of the pretrained demonstrator. From this point onward, the process can drift equally in either direction.

Based on our observations, it was clear to us that naïve group members were motivated to approach the foraging grid and to attempt to open the covers only after the demonstrators did it first. Thus, it is most likely that the demonstrators provided social motivation (or social facilitation) to other group members. However, after being motivated to try and open covers, the task was possibly easy enough to allow some innovations by naïve individuals (as well as by some of the demonstrators themselves), and these innovations shifted information transfer to the other direction than the seeded one. Interestingly, this initial flexibility in learning dynamics and information transfer is in sharp contrast to the persistence exhibited by both demonstrators and naïve individuals after they already acquired one of the methods (Fig. 5). This contrast is especially pronounced by the pretrained demonstrators who continued to prefer their method even after most of their group members used the other one (see groups NIS, THG, KOT, and HVR in Fig. 4). Thus, learning dynamics and information transfer in the group are initially flexible and prone to be affected by chance events when naïve birds are just acquiring the skill, but become relatively fixed after each bird adopts its preferred method (see Pesendorfer et al. 2009; Van de Waal et al. 2013 for similar results). We cannot determine how long the babblers can remember and persist with their cover-opening method; this question remains open for further research. However, it is worth noting that similar field studies of wild marmosets have demonstrated that behavioral fidelity following a 2-action setting may last for many months, and even years (Gunhold et al. 2014, 2015).

There has been much interest recently in how groups can change their preacquired culture following innovations, and how such changes may be facilitated by population turnover and by naïve individuals (Aoki et al. 2011; Shizuka and Johnson 2020). It has been suggested that naïve individuals may be better at freely exploring the relative value of alternative behaviors based on individual learning, which allows them to adopt a novel and more beneficial behavior and demonstrate it to the rest of the group (Chimento et al. 2021; Chimento and Aplin 2024). Our study suggests that even when there are no major differences in the value of alternative task solutions, naïve group members may play a critical role in shaping group culture when innovations appear. That is, a novel behavior introduced, eg into 2 different groups (eg opening covers by lifting) may not necessarily be copied as originally demonstrated but may elicit a slightly different innovation by the first naïve learners in one of the groups (eg opening covers by pecking rather than by lifting), leading to different cultures in those different groups.

## Supplementary Material

araf099_Supplementary_Data

## Data Availability

Analyses reported in this article can be reproduced using the data provided by Aljadeff et al. (2025).
