# Supplementary material for: Complex dynamics of social learning in groups of wild Arabian babblers
Source: Behav Ecol. 2025 Sep 15;36(5):araf099. doi: 10.1093/beheco/araf099 (PMC12477425; doi:10.1093/beheco/araf099)
Supplement: araf099_Supplementary_Data [file araf099_supplementary_data.docx]

**Supplementary materials**

**
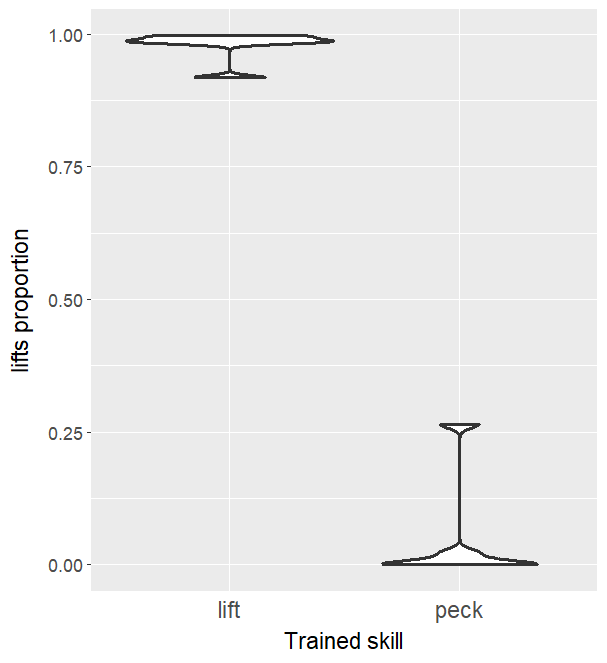
**

**Figure S1**

Probability density plot of the lift proportion exhibited by pre-trained demonstrators during all sessions of Stage 2 shows consistent behavior aligned with their training, with lift demonstrators (*n*=6) scoring around 1 and peck demonstrators (*n*=6) scoring close to zero. The opening scores of both groups significantly deviated from the 0.5 scores expected at random (Binomial exact test, n=6, p<0.001 in both cases).

a) b)


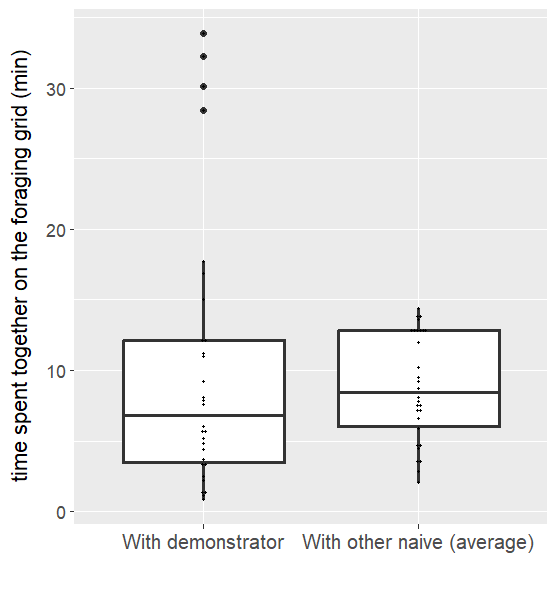


**Figure S2**

The duration of social foraging of focal naïve birds with a) the group's pre-trained demonstrator, and b) other naïve group members, during all sessions of stage 2. The duration spent with the pre-trained demonstrator (10.2±9.6 minutes) did not significantly differ from the average time spent with other naïve individuals (8.9±3.8 minutes; Wilcoxon paired signed-rank test, n=30, p>0.6). Data are represented as data points, median, 25%, and 75% quantiles. Not shown: Demonstrators exhibited a higher mean cover opening rate (11.7±2.1 openings per minute) compared to naïve individuals (3.7±3.9 openings per minute), who also showed greater variability in their performance.


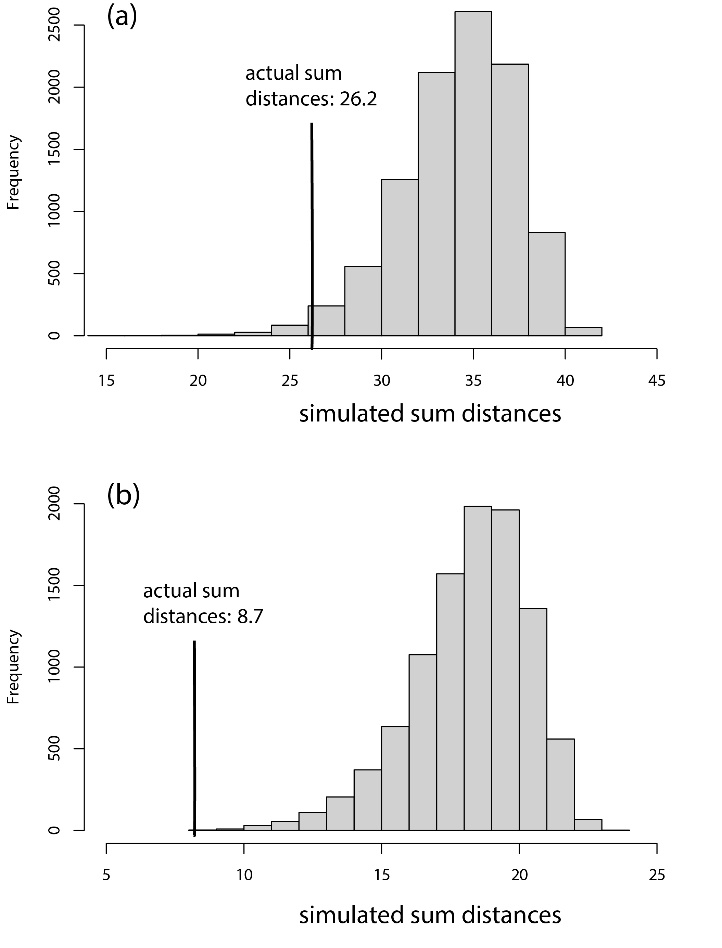


**Figure S3**

Histograms depicting the null distribution of the sum of within-group differences expected by chance. Generated through a permutation test with lift proportion scores randomly assigned to simulated groups, representing the original experiment's group sizes. The actual sum of differences shows the observed sum of within-group differences in lift proportion scores of birds in the experiment when (a) including the pre-trained demonstrators (44 individuals - naïve and demonstrators - from 12 groups, estimated p-value = 0.0143, effect size= -7.99, 95% CI [27.18 - 39.10]), and (b) only naïve individuals included (30 naïve individuals from 10 groups, estimated p-value = 0.0001, effect size= -9.5, 95% CI [13.22 - 21.49]). P-Values were estimated based on the number of simulated sums of differences below the observed sum of differences, divided by the number of simulation iterations.

**
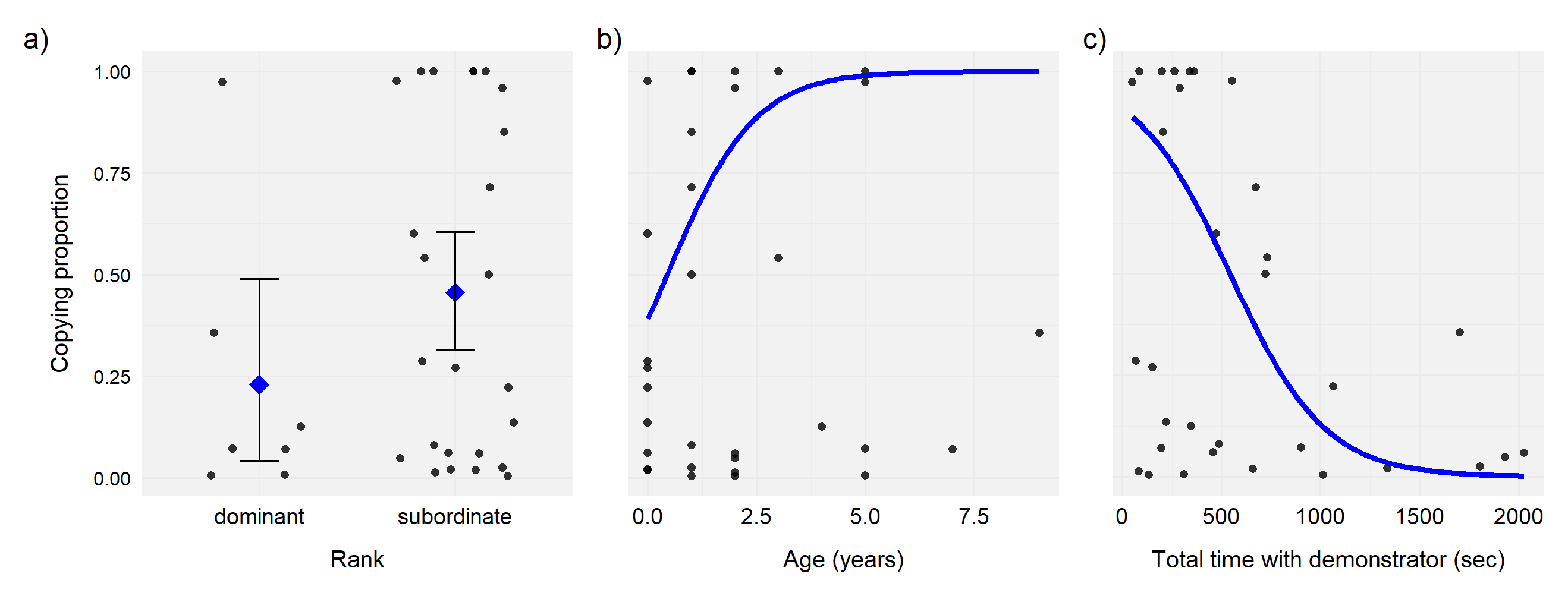
Figure S4**

Effects of individual rank, age and time spent with the demonstrator on copying behavior, based on the GLMM of Table S2. Points represent individual data (jittered in (a) to reduce overlap), blue points and error bars show model predicted means and 95% confidence intervals, and blue lines represent model-predicted relationships. a) Copying proportion was significantly higher among subordinate individuals. b) A positive relationship was found between age and copying proportion, indicating that older individuals within each rank class were more likely to copy the demonstrator. c) Copying proportion decreased with time spent with the demonstrator on the foraging grid.

**
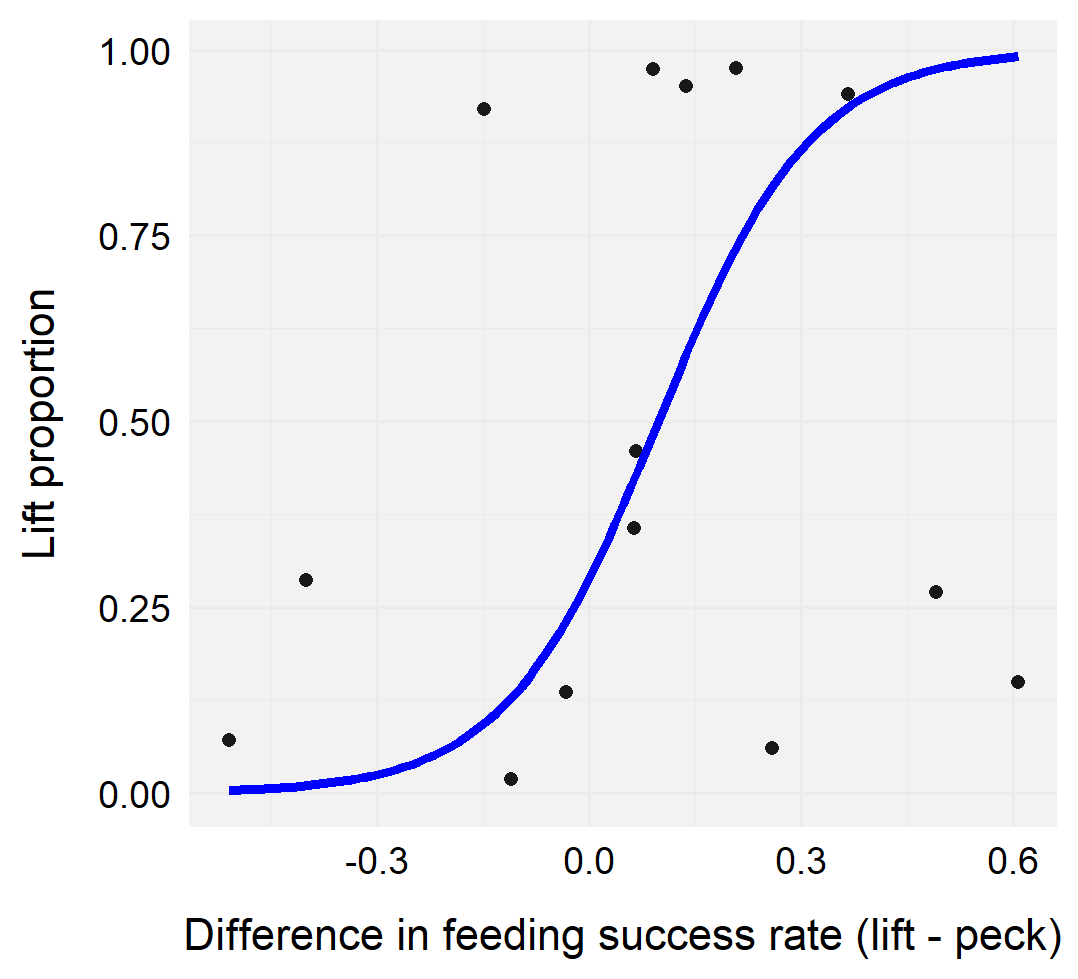
**

**Figure S5**

Relationship between relative feeding success and lift preference predicted by the GLMM of Table S3 (points represent individual data, and the blue line shows the predicted relationship).

**
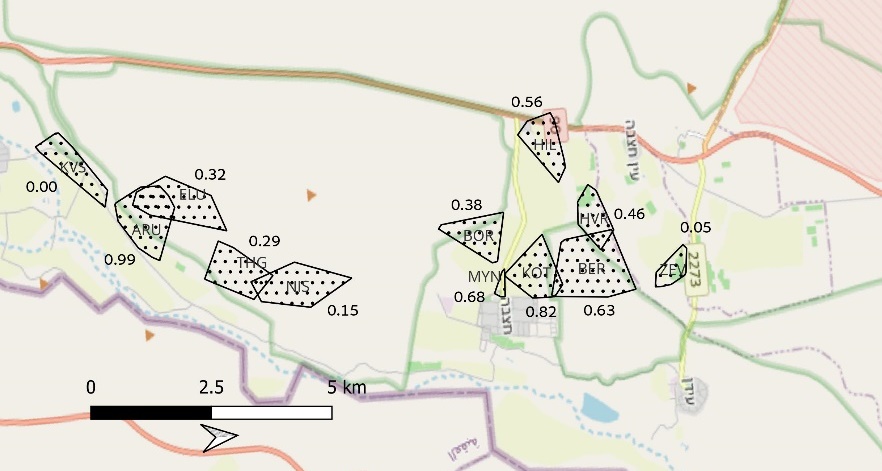
**

**Figure S6**

Geographic distribution of the 12 group territories, with numbers indicating the lifting proportion of naïve individuals in each group. Groups with close territories show varying lifting proportions, suggesting environmental conditions cannot explain the differences. Territory data were collected by researchers using the Anecdata app and downloaded from the Arabian Babbler Survey. Spatial analyses and map visualizations were conducted in QGIS (2024), with territories delineated using the Minimum Bounding Geometry algorithm.

**Table S1**

The number of naïve individuals in each babbler group that did not learn to solve the task, solved it and performed less than six foraging steps, and solved it and performed six foraging steps or more.


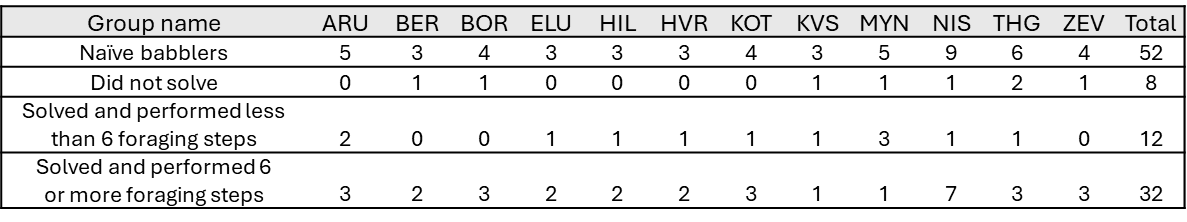


**Table S2**

Results of GLMMs testing the effects of additional individual and group characteristics on copying behavior. Model A includes all tested predictors; Model B includes only those with significant effects.

| Model | N | Response variable | Fixed effects | Estimate | Std. error | z value | p value |
| --- | --- | --- | --- | --- | --- | --- | --- |
| A | 30 | Copying success of demonstrator by naïve individuals | (Intercept) | 2.976 | 8.046 | 0.370 | 0.711 |
|  |  |  | demonstrator skill (peck) | 0.736 | 1.400 | 0.526 | 0.599 |
|  |  |  | Observed lift proportion of demonstrator by naïve individuals: Group means | -1.420 | 7.362 | -0.193 | 0.847 |
|  |  |  | **Observed lift proportion of demonstrator by naïve individuals: Deviations from group means** | 43.770 | 5.783 | 7.569 | **<0.001** |
|  |  |  | **Rank of naïve individuals (subordinate)** | 2.645 | 0.716 | 3.693 | **<0.001** |
|  |  |  | **Age of naïve individuals** | 0.961 | 0.139 | 6.912 | **<0.001** |
|  |  |  | Sex of naïve individuals (M) | 0.049 | 0.274 | 0.179 | 0.858 |
|  |  |  | Number of group members | -0.827 | 0.555 | -1.491 | 0.136 |
|  |  |  | Demonstrator rank | 0.743 | 1.546 | 0.480 | 0.631 |
|  |  |  | Demonstrated color (orange) | 0.757 | 1.368 | 0.553 | 0.580 |
|  |  |  | Total time with demonstrator | -0.004 | 0.000 | -13.710 | <0.001 |
| B | 32 | Copying success of demonstrator by naïve individuals | (Intercept) | -1.631 | 0.858 | -1.901 | 0.057 |
|  |  |  | Observed lift proportion of demonstrator: Deviations from group means | 42.176 | 4.428 | 9.525 | <0.001 |
|  |  |  | Rank of naïve individuals (subordinate) | 2.902 | 0.549 | 5.285 | <0.001 |
|  |  |  | Age of naïve individuals | 0.999 | 0.097 | 10.339 | <0.001 |

**Table S3**

Results of a GLMM testing whether naïve individuals’ preference for the lift method was related to their relative feeding success with lifting versus pecking.

| *N* | Response variable | Fixed effects | Estimate | Std. error | z value | p value | 95% CI  (lower, upper) |
| --- | --- | --- | --- | --- | --- | --- | --- |
| 14 | Lift proportion by naïve individuals | (Intercept) | -0.901 | 1.196 | -0.753 | 0.451 | (-3.57, 1.75) |
|  |  | Difference in success rate (lift - peck) | 9.216 | 0.573 | 16.088 | <0.001 | (8.12, 10.37) |
|  |  |  |  |  |  |  |  |
